# Dynamic choice HIV prevention with long-acting injectable cabotegravir pre-exposure prophylaxis in east, central, southern, and west Africa: a cost-effectiveness modelling analysis

**DOI:** 10.1016/S2352-3018(25)00169-9

**Published:** 2025-09-11

**Authors:** Andrew N Phillips, Matthew D Hickey, Starley B Shade, Jane Kabami, James Ayieko, Paul Revill, Elijah Kakande, Laura B Balzer, Nicole Sutter, Loveleen Bansi-Matharu, Jennifer Smith, Gabriel Chamie, Diane V Havlir, Moses R Kamya, Maya Petersen

**Affiliations:** Institute for Global Health, UCL, London, UK (Prof A N Phillips PhD, L Bansi-Matharu PhD, J Smith PhD); Division of HIV, Infectious Diseases and Global Medicine, Department of Medicine, University of California, San Francisco, CA, USA (M D Hickey MD, S B Shade PhD MPH, N Sutter MPH, Prof G Chamie MD, Prof D V Havlir MD); Institute for Global Health Sciences, Department of Epidemiology and Biostatistics, University of California, San Francisco, CA, USA (S B Shade); Infectious Diseases Research Collaboration, Kampala, Uganda (J Kabami MPH, E Kakande MD); Kenya Medical Research Institute, Nairobi, Kenya (J Ayieko PhD); University of York, York, UK (P Revill MSc); Division of Biostatistics, School of Public Health, University of California, Berkeley, CA, USA (L B Balzer PhD, Prof M Petersen MD PhD); School of Medicine, Makerere University, Kampala, Uganda (Prof M R Kamya PhD)

## Abstract

**Background:**

In randomised controlled trials in Kenya and Uganda, a dynamic choice HIV prevention (DCP) intervention that offered structured choice of biomedical prevention product and opportunity to change products over time substantially improved prevention coverage; incident HIV infections were eliminated when long-acting cabotegravir was included as an option. We aimed to assess the potential cost-effectiveness of the intervention regimen in east, central, southern, and west Africa.

**Methods:**

We used the existing individual-based HIV Synthesis model. Through sampling of parameter values at the start of each model run of a simulated population of adults, we created 1000 setting-scenarios, reflecting uncertainty in assumptions and a range of characteristics similar to those seen in east, central, southern, and west Africa. For each setting-scenario, we simulated predicted outcomes including disability-adjusted life-years (DALYs) and costs up to 50 years resulting from (1) continuing with the status quo (ie, no DCP); (2) introduction of the DCP intervention with oral pre-exposure prophylaxis (PrEP), post-exposure prophylaxis (PEP), and condoms without long-acting cabotegravir PrEP (ie, DCP without cabotegravir); and (3) introduction of the DCP intervention and including long-acting cabotegravir PrEP (ie, DCP including cabotegravir). We used a cost-effectiveness threshold of US$500 per DALY averted, and a discount rate of 3% per year. The annual cost of DCP including cabotegravir was assumed to be $190 per person. Net DALYs averted was calculated by DALYs averted plus the difference in costs divided by the cost-effectiveness threshold.

**Findings:**

Reflecting the trial results, among people with a PrEP indication (ie, having an HIV acquisition risk) and an HIV test in the past 3 months, the median proportion of people on PrEP was 14% (90% range 4–43) with no DCP, 54% (23–74) with DCP without cabotegravir, and 71% (35–83) with DCP including cabotegravir. These increases in PrEP use led to HIV incidence reductions, with incidence rate ratios of 0·89 (0·67–1·17) for DCP without cabotegravir and 0·64 (0·44–0·97) for DCP including cabotegravir, relative to no DCP. Across setting-scenarios, both DCP policies led to DALYs being averted: 18 400 DALYs per year (95% CI 16 700–20 100) for DCP including cabotegravir and 56 400 DALYs per year (52 300–60 500) for DCP without cabotegravir in 10 million adults. Compared with no DCP, there was a mean increase in annual discounted costs over 50 years: $8·6 million (7·7–9·4) for DCP without cabotegravir and $13·2 million (11·6–14·8) for DCP including cabotegravir. Addition of long-acting cabotegravir PrEP to DCP was cost-effective (*vs* DCP without cabotegravir); the incremental cost-effectiveness ratio for DCP including cabotegravir (*vs* no DCP) was $234 per DALY averted. There was substantial variation across setting-scenarios and we found that DCP was more likely to be the cost-effective choice in settings with high prevalence of unsuppressed HIV or low proportion of people with an indication for PrEP.

**Interpretation:**

Offering structured PrEP and PEP choice including long-acting cabotegravir and enabling risk-informed use could reduce HIV incidence by a third over 10 years. If projected generic production costs of long-acting cabotegravir can be realised, it is likely to be cost-effective across multiple settings in east, central, southern, and west Africa.

**Funding:**

US National Institutes of Health.

## Introduction

HIV incidence in east, central, southern, and west Africa has declined over the past 20 years but remains high.^1^ There is a need to strengthen primary HIV prevention. Three randomised controlled trials in rural Kenya and Uganda evaluated a dynamic choice HIV prevention (DCP) approach developed by the SEARCH team.^2–5^ These trials involved offering integrated pre-exposure prophylaxis (PrEP) with daily oral tenofovir disoproxil fumarate plus emtricitabine and post-exposure prophylaxis (PEP) to both men and women with self-assessed risk for HIV acquisition in a range of settings, including primary health centre outpatient departments, antenatal clinics, and in the community with delivery facilitated by community health workers. The intervention (DCP) included provider training in offering choices between biomedical prevention products, with the option to change over time, prevention counselling, choice of visit location (home or clinic) and HIV testing modality (rapid antibody or self-test), person-centred delivery, including telephone access to a provider, and structured assessment of, and response to, prevention uptake barriers. DCP led to substantial benefits in biomedical prevention coverage (proportion of follow-up time using PrEP or PEP) compared with standard-of-care biomedical prevention services.^2–5^

Long-acting injectable cabotegravir PrEP, which leads to a substantial reduction in HIV acquisition risk,^6^ was not initially available for inclusion in the described trials, but subsequently became available. In a 48-week extension study to the three trials, long-acting cabotegravir PrEP was added as an additional prevention product choice for participants in the DCP group. Participants were about 75% female, with around a third aged 15–24 years and the rest of the participants being older than 24 years. The authors hypothesised that an oral PrEP, PEP, and long-acting cabotegravir PrEP biomedical prevention package with structured choice between products and opportunities to switch would increase biomedical prevention coverage compared with standard-of-care among men and women at risk for HIV. The trial results indicated that mean biomedical covered time was higher with DCP (69·7%) than with standard-of-care (13·3%), with a difference of 56·4% (95% CI 50·8–62·1; p<0·0001); incidence of HIV was 0% with DCP including long-acting cabotegravir PrEP compared with 1·8% with standard-of-care (p=0·014).^7^

Cost-effectiveness of the DCP approach in the context of long-acting cabotegravir PrEP availability has not previously been evaluated. We used an existing individual-based model of HIV transmission and the effect of antiretroviral drug-based prevention and therapy to evaluate the potential cost-effectiveness of this approach in east, central, southern, and west Africa.

## Methods

### Model design

We used the previously described^8–10^ HIV Synthesis model (appendix pp 16–126). Each model run generated a simulated population of 100 000 adults (aged 15 or older) from 1989 (taken as the start of the epidemic) to 2075 (50 years after intervention introduction) with variables updated every 3 months, including age, sex, primary and non-primary condomless sex partners, whether currently a female sex worker, HIV testing, male circumcision status, presence of sexually transmitted infections other than HIV, and use of oral and cabotegravir PrEP. Only heterosexual sex was modelled. In people with HIV, we modelled viral load, CD4 cell count, use of specific antiretroviral drugs, and drug resistance. Risk of AIDS death in the model depended on CD4 cell count, viral load, age, and antiretroviral therapy (ART) status. Presence of drug resistance to individual drugs was considered, including resistance mutations arising in people who were on PrEP but living with HIV.^10^

By sampling of parameter values (appendix p 83) at the start of each model run, we created 1000 setting-scenarios, reflecting uncertainty in assumptions and a range of characteristics similar to those seen in settings within east, central, southern, and west Africa (table 1). We incorporated more uncertainty and variability than might be seen by considering specific available published estimates only. We compared characteristics of settings with country data from Population-based Health Impact Assessment surveys, but the aim was to reflect subsettings within countries as well as countries as a whole. We presented results in terms of median and 90% range across setting-scenarios.

For each setting-scenario, we simulated predicted outcomes resulting from three policies: (1) continuing with the status quo, with some oral PrEP use and free condom availability, but no PEP or long-acting cabotegravir PrEP (ie, no DCP); (2) introduction of the DCP intervention with oral PrEP, PEP, and condoms (informed by results of the initial DCP trials;^2–5^ ie, DCP without long-acting cabotegravir PrEP); and (3) introduction of DCP intervention including long-acting cabotegravir PrEP (informed by the results of the long-acting cabotegravir extension of these trials; ie, DCP including cabotegravir).^7^

Details of modelling of oral PrEP and cabotegravir-PrEP have been previously described^8,10^ (appendix p 44). In brief, PrEP (and, by definition, PEP) use was assumed to be risk-informed and a person was defined as having an indication for PrEP in a given 3 month period if they had condomless sex with at least one short-term partner or a long-term partner known to have HIV but not on ART, or when a woman felt there was a high risk that her long-term partner was in this position (implemented as women younger than 50 years without HIV with a long-term condomless sex partner who was not on ART). We considered five variations, as described in the appendix (p 44). For all policies, people not at risk were assumed not to take PrEP. As shown in table 1, our assumptions on PrEP uptake and persistence meant that at baseline the proportion of people with an indication for PrEP who were taking oral PrEP had a median of 5·2% across setting-scenarios (90% range 1·7–16·6).

Prevention effectiveness of oral PrEP in a given 3-month period depended on efficacy and pill-taking adherence among persisting users. Efficacy of oral PrEP (ie, with 100% adherence and against tenofovir and lamivudine-sensitive virus) was assumed to be 95% (80% chance) or 90% (20% chance) and for each model run we selected randomly from the two, with an 80% chance of 95% as it was considered to be more likely.^11^ Efficacy of long-acting cabotegravir PrEP (against cabotegravir-sensitive virus) was assumed to be 90% (20% chance), 95% (40% chance), or 98% (40% chance).^6^ PEP use was also dependent on adherence and we modelled PEP efficacy and adherence to be the same as for oral PrEP, and we did not explicitly differentiate whether a person was on PrEP or covered by PEP in a given 3-month period.

Modelling of DCP was based on results from the DCP trials (appendix p 82).^2–5,7^ Under the two DCP policies, we assumed that all PrEP and PEP for HIV prevention was delivered under a DCP care model. The indication for DCP was assumed to be the same as the indication for PrEP. While on DCP, a person could receive oral PrEP, PEP, or (if in the DCP including cabotegravir group) long-acting cabotegravir, or could receive prevention services without biomedical HIV prevention and make visits to test and discuss prevention. People on PrEP were tested for HIV every 3 months. For those on DCP but not on PrEP or PEP, there was an elevated chance of testing for HIV and of starting PrEP or PEP compared with if they were not under DCP due to ongoing visits to discuss PrEP and PEP and choice regarding product and delivery options including visit location. There was also a reduced chance of stopping PrEP (despite continued indication) compared with if the DCP intervention was not in place. In sum, this resulted in higher coverage of PrEP and PEP during periods of risk under DCP (with or without long-acting cabotegravir PrEP) compared with no DCP, reflecting DCP trial results.^2–5,7^

A proportion of HIV tests were assumed to be self-tests (appendix p 44). Under DCP we considered potential for greater use of self-tests than for no DCP based on DCP trial results. In the model, individuals under DCP who no longer had an indication for PrEP or DCP stayed in the DCP group for 3 months and then exited DCP care. There was also a specified rate of stopping DCP (despite continued indication for PrEP and DCP; appendix p 82).

### Cost-effectiveness analysis

We assessed the effects of the policies, beginning in 2025, over 10 years and 50 years to assess long term effectiveness (disability-adjusted life-years [DALYs]) and cost-effectiveness. 10-year outcomes were assessed as there is wide interest in these short-term effects, while the long-term effects were assessed to make our cost-effectiveness analysis appropriately comparable with other such evaluations in which considering a long time horizon to fully take into account all consequences of introducing the various policies under consideration is important. Absolute numbers of health-related events, costs, and DALYs are relevant for a setting or country of population size of 10 million adults in 2024. Cost-effectiveness analysis was conducted from a health-care perspective, costs and health outcomes were both discounted at 3% per annum, and a cost-effectiveness threshold of US$500 per DALY averted was used. Country-specific thresholds are uncertain but $500 per DALY averted is likely to be at the upper end on the basis of evidence concerning how resources would otherwise be used,^12^ particularly in the increasingly constrained donor funding environment. We used this threshold to calculate net DALYs averted.^13^ Net DALYs take into account the health consequences of the difference in costs and the difference in health and reflect the impact of a policy on overall population burden of disease. Net DALYs averted was calculated by DALYs averted plus the difference in costs divided by the cost-effectiveness threshold. We also modelled pregnancies, births, and periods of breastfeeding for each woman, with the probability of transmission dependent on the mother’s viral load (appendix p 21). For each child infected through mother-to-child transmission, we assumed that 5 DALYs (with discounting) were incurred; given the death rate from HIV in children living with HIV,^14^ this assumption was probably an underestimate of the DALYs incurred, resulting in a conservative estimate of DCP and PrEP benefits. For our results related to cost-effectiveness over the 50 year time horizon, we present results as the mean over setting-scenarios and the 95% CIs for the mean to convey the average effect and uncertainty over setting-scenarios due to stochastic effects, as well as the median and 90% range across setting-scenarios.

Clinic costs for people on oral PrEP were assumed to be $10 per 3 months, with a drug cost of $15 per 3 months.^15^ PEP cost was uncertain as it was dependent on the number of times PEP was used in a 3-month period, and because PEP contains an extra drug, dolutegravir, compared with oral PrEP. We conservatively assumed a similar cost for PEP per 3 months as oral PrEP. For long-acting cabotegravir, we assumed clinic costs of $15 per 3 months and drug costs of $15 per 3 months. The drug cost was based on assuming generic production^16^ and, recognising the uncertainty in this assumption, we considered alternative values. We accounted for the cost of the more frequent 2-monthly rather than 3-monthly testing. Clinic costs per 3 months per individual under the DCP intervention were assumed to be $12, in addition to PrEP clinic and drug costs and costs of testing. These DCP cost estimates were based on trial data and account for additional time, provider training, and telephone support required to implement the DCP intervention compared with standard PrEP care (appendix p 106). Thus, the cost of DCP including cabotegravir was $15 per 3 months for long-acting cabotegravir drug ($60 per year), $15 per 3 months for long-acting cabotegravir injection administration (clinic cost; $60 per year), $12 per 3 months for a DCP visit ($48 per year), and $22 for six HIV tests per year, totalling $190 for a person on DCP including cabotegravir for a full year.

Costs for tenofovir–lamivudine–dolutegravir for people with HIV were assumed to be $50 per year including supply chain costs.^17^ Clinic costs for people with HIV were assumed to be $10 per 3 months if the person was not known to have viral suppression and $5 per 3 months if the person had a viral load measure showing viral suppression in the past year.^18^ The lifetime cost incurred, with discounting, as a result of each child born with HIV was assumed to be $1000, which was probably a substantial underestimate,^19^ but we wished to err on the conservative side in our evaluation of the DCP intervention. Other costs and disability weights are shown in the appendix (pp 106–7).

### Role of the funding source

The funder of the study had no role in study design, data collection, data analysis, data interpretation, or writing of the report.

## Results

Without DCP the proportion of people with a PrEP indication who took PrEP was low (table 2). DCP without cabotegravir availability increased the proportion of people with a PrEP indication (ie, at risk for HIV acquisition, reflected in trial data) who took PrEP; inclusion of long-acting cabotegravir in DCP led to an additional increase due to the higher willingness (reflected in trial data) of individuals to take long-acting cabotegravir PrEP if offered compared with oral PrEP and PEP. With the DCP policies, the proportion of people under DCP who had a PrEP indication in any one 3-month period did not differ according to whether long-acting cabotegravir PrEP was available (table 2). This increased used of PrEP in those with an indication means there was an increase in the overall proportion of adults aged 15–64 years who were on PrEP with DCP and a further increase with the addition of long-acting cabotegravir (table 2). A high proportion of people on PrEP under DCP in the context of long-acting cabotegravir availability take long-acting cabotegravir (77%) rather than oral PrEP.

Increases in PrEP use led to HIV incidence reductions relative to the no DCP policy, with greater reductions with the addition of long-acting cabotegravir than with DCP with no long-acting cabotegravir (table 3). This lower incidence was predicted to result in a slightly higher percentage of people positive for HIV diagnosed, a higher percentage of all people positive for HIV with viral loads less than 1000 copies per mL, a lower prevalence of HIV viral load more than 1000 copies per mL among all adults, and a lower overall HIV prevalence. The percentage of children born to mothers with HIV who had HIV by the end of the breastfeeding period was also lower with the DCP intervention, particularly with the inclusion of long-acting cabotegravir PrEP.

Additional costs due to higher PrEP use and implementation of DCP are somewhat compensated by cost reductions resulting from fewer people living with HIV and requiring care (table 4; appendix p 2).

Overall, as a mean across setting-scenarios, both DCP policies lead to DALYs being averted (table 5). There was, however, variability over setting-scenarios (appendix p 3); the policy with the least DALYs incurred was the status quo (no DCP or long-acting cabotegravir) in 6% of setting-scenarios, DCP without cabotegravir in 11% of setting-scenarios, and DCP including cabotegravir in 83% of setting-scenarios.

Compared with the status quo, as a mean across setting-scenarios, there was an increase in annual discounted costs over 50 years for the policy of DCP without cabotegravir of $8·6 million (95% CI 7·7–9·4) and for the policy of DCP including cabotegravir of $13·2 million (11·6–14·8). There was variability across setting-scenarios.

The policy of DCP without cabotegravir was not a candidate for the cost-effective choice of intervention, as a combination of the policies of status quo and DCP including cabotegravir (represented by a point half way along the line between the two policies) was more effective and less costly than this policy (ie, the policy was extendedly dominated). The incremental cost-effectiveness ratio (ICER) for the policy of DCP including cabotegravir was $234 per DALY averted (figure). This policy incurred the lowest overall mean net DALYs at a cost-effectiveness threshold of $500 per DALY averted (table 5). If long-acting cabotegravir was not available, the ICER for DCP was $467.

The policy that resulted in the least net DALYs incurred (ie, was cost-effective in the context of a cost-effectiveness threshold of $500 per DALY averted) was no DCP in 30% of setting-scenarios, DCP without cabotegravir in 14%, and DCP including cabotegravir in 56% of setting-scenarios. Thus, DCP in one form or the other was cost-effective in 70% of setting-scenarios.

We assumed a cost of DCP of $12 per person per 3-month period on the basis of trial data, additional to the clinic and drug costs of providing PrEP and including for people under DCP but not on PrEP. The ICER remained less than $500 so long as this additional DCP care delivery cost was less than $25 (table 5). Also shown is the ICER according to differences in the annual per person cost of long-acting cabotegravir PrEP drug. We assumed $60 in our main analysis but the ICER for DCP including cabotegravir remained less than $300 per DALY averted when this cost was $80.

Across setting-scenarios, the proportion of people with an indication for PrEP and the prevalence of unsuppressed HIV at baseline in 2024 were two strong predictors of DCP including cabotegravir being cost-effective (appendix p 4). For an annual cost of DCP including cabotegravir of $190 (used in our main analysis), the ICER for DCP including cabotegravir was more than $500 if the proportion of people aged 15–64 years with an indication for PrEP was more than 4% and the prevalence of unsuppressed HIV was less than 2% or if the proportion of people aged 15–64 years with an indication for PrEP was more than 7% and the prevalence of unsuppressed HIV was less than 4% (table 6). When the prevalence of unsuppressed HIV was more than 2%, the ICER can be less than $100 per DALY averted. Even with a higher annual cost of DCP including cabotegravir of $330, DCP including cabotegravir was cost-effective based on a $500 per DALY averted threshold if the proportion of people aged 15–64 years with an indication for PrEP was low and the prevalence of unsuppressed HIV more than 2%.

Patterns of sexual behaviour influenced cost-effectiveness, as seen with the influence of the proportion of people aged 15–64 years with an indication for PrEP (appendix pp 6–11). The rate of stopping long-acting cabotegravir PrEP for people on DCP was the most influential parameter on DALYs and cost-effectiveness (appendix pp 12–15).

## Discussion

Offering structured PrEP and PEP choice including long-acting cabotegravir enabled risk-informed use and could reduce HIV incidence by a third over 10 years. If projected generic production costs of long-acting cabotegravir can be realised it is likely to be cost-effective across multiple settings in east, central, southern, and west Africa.

Effective approaches for delivering biomedical HIV prevention to meet the diverse and dynamic needs of both women and men, including in rural settings, are urgently needed. The SEARCH DCP trials have provided evidence on the benefits of a choice-based person-centred model for delivering HIV prevention in real-world settings, including in the context of incorporating long-acting cabotegravir.^2–5,7^ We evaluated the potential cost-effectiveness of introducing DCP intervention, and found that the policy of DCP including cabotegravir was likely to be a cost-effective policy choice in multiple settings, if long-acting cabotegravir could be sourced at around $80 per year or less. There is potential for cabotegravir to be produced at a cost less than $20 per year if there is high demand.^16^ We assessed the DCP intervention in the context of a wide range of setting-scenarios that reflected the variability across settings within east, central, southern, and west Africa. We found that DCP was more likely to be the cost-effective choice in settings with high prevalence of unsuppressed HIV and settings with low proportion of people with an indication for PrEP. Although survey data, such as PHIA surveys, can provide information on the former, the latter might be difficult to ascertain for a setting as it relies on accurate self-report of sexual behaviour.

As previously published,^8,10^ in all policies considered we assumed PrEP use was risk-informed and selectively used during periods condomless sex with new partners and not used for extended periods of low risk. Being under DCP is also risk-informed, in that those who enrol have a self-assessed risk for HIV acquisition. Although this self-assessed risk was successfully done in the trials in Kenya and Uganda, in practice in some settings it might present a challenge to reach and engage with those with a self-assessed risk. Results from implementation in rural communities in the SEARCH study were encouraging and show that high pill-taking adherence to PrEP in persisting users during periods of risk can lead to substantial declines in incidence.^20,21^ In respect of our assumption of close alignment of periods of risk with periods of PrEP use, our modelling differs to some extent from other modelling of long-acting PrEP, which does not always show PrEP to be cost-effective.^22–26^

In our modelling we captured the observed effects of the DCP intervention. For example, in the DCP trial, coverage of periods of risk with biomedical prevention was estimated to be 70% with DCP plus long-acting cabotegravir PrEP, compared with 13% in the control group, which was reflected in our model in that the proportion of people with a PrEP indication and an HIV test in the past 3 months on PrEP was 14% (90% range 4–43), increasing to 71% (35–83) with DCP including cabotegravir. Nevertheless, we might have underestimated or overestimated the benefits of the DCP intervention. For example, effects could be attenuated at scale or over extended time horizons; or they could be enhanced due to broader awareness of DCP and, therefore, have greater population-level reach among those at risk. We projected an incidence rate ratio of 0·66 with DCP including cabotegravir compared with no DCP. Similarly in the trial, there were no new acquisitions over 48 weeks of follow-up under DCP including cabotegravir, compared with seven in the standard-of-care group (incidence rate 1·8 per 100 person-years; incidence rate ratio 0·00).^7^

Our modelling reflected observed costs, which could overestimate or underestimate the costs of the intervention in a given setting (including both costs for oral and long-acting cabotegravir PrEP) at scale and over time. These estimates could be affected by health-system integration, delivery support by community health workers and other lay health providers, and economies of scale: if PrEP costs are higher than those used here in a given setting interventions will be less likely to be cost-effective. We estimated costs of delivering DCP on the basis of observed trial costs, although additional costs might be required to sustain implementation in routine settings. In sensitivity analyses, we estimated that DCP would be cost-effective even if implementation costs were double what we estimated. Although DCP visits were not substantially longer than standard HIV prevention visits, opportunity costs of implementing DCP might be greater in settings where health-care workers are already overburdened with delivery of additional evidence-based interventions and do not have capacity for additional intervention delivery.

Another long-acting injectable drug, the capsid inhibitor lenacapavir, has extremely high efficacy in reducing risk of HIV acquisition.^27^ Lenacapavir injections are required every 6 months and are subcutaneous. Our results on DALY benefits would probably hold true were lenacapavir the long acting PrEP drug rather than cabotegravir. Indeed, the DALY benefit could be slightly greater with lenacapavir because cabotegravir is a drug with cross resistance with dolutegravir, which is widely used for treatment.^10^ However, the tolerability of lenacapavir outside the phase 3 trial setting is unknown. Assuming lenacapavir tolerability results in similar uptake, the relative cost-effectiveness of the two drugs is likely to depend on their relative costs.

We sampled parameter values from distributions that reflected both uncertainty and variability between settings. As shown, the approach allowed us to identify the key features of an epidemic in a setting that predicted effectiveness and cost-effectiveness of the policies of interest. We acknowledge as a limitation, however, that this approach means we cannot be certain that each setting-scenario has relevance to a setting in east, central, southern, and west Africa. Traditionally, a cost-effectiveness analysis might often be based on one specific country or setting, which allows particular emphasis on reflecting that setting as closely as possible. This approach does, however, have the disadvantage of offering guidance for only that specific country or setting and not allowing explorations to help understand drivers of impact and cost-effectiveness more broadly for countries in a given region.

Additionally, our approach to determining distributions for parameter values tended to consider more variability and uncertainty than is reflected in specific data points from published research, which we consider to be appropriate as the range of published estimates is unlikely to reflect the full range of uncertainty and variability. This uncertainty was propagated through to our policy question results when we expressed percentages of setting-scenarios in which a policy was cost-effective.

Further, we only considered heterosexual sex. Our model’s 3-month time step means that we approximated the effects of cabotegravir over a 2-month time step, although we took into account the extra costs that result from more frequent visits and testing. We recognise that modelling PEP not distinguished from PrEP is a limitation. The 3-month time step for our model contributes to this limitation, since PEP is a 28-day course. We also note that there are no definitive estimates of PEP efficacy. Finally, for each sampled set of parameter values we ran the model just one time rather than multiple times and taking the mean.

In conclusion, the DCP intervention has a high probability of being cost-effective in multiple settings should it be possible to source long-acting HIV prevention drugs at sufficiently low costs.

## Supplementary Material

Appendix

## Figures and Tables

**Figure:**
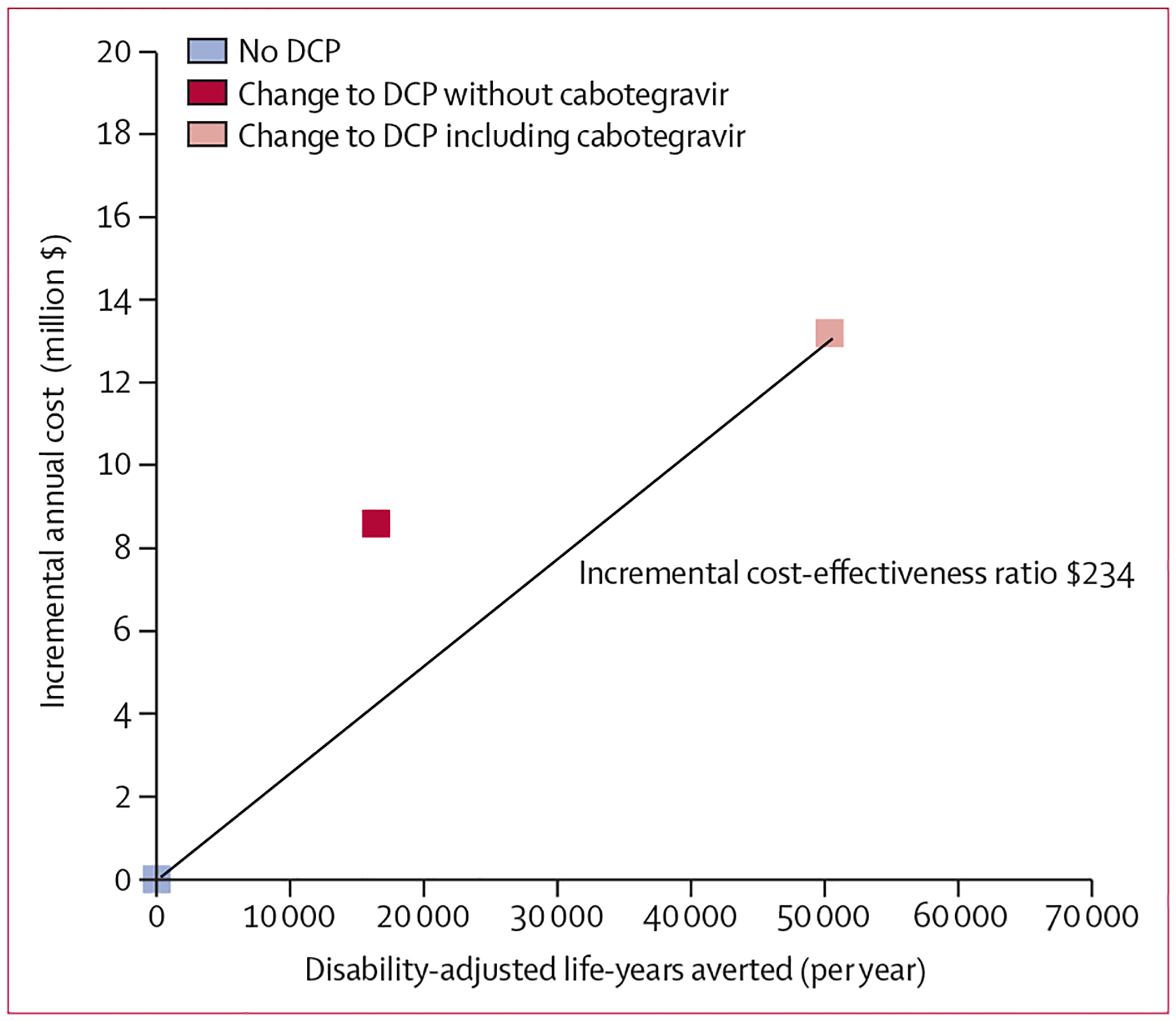
Cost-effectiveness analysis; differences in disability-adjusted life-year and costs and incremental cost-effectiveness ratio Monetary values are US$. DCP=dynamic choice HIV prevention.

**Table 1:** Description of setting-scenarios in 2024 based on 1000 setting-scenarios

	Model	Examples of observed data*
HIV prevalence in women aged 15–49 years	14·7% (4·5–45·3)	Zimbabwe 2020 15%; Tanzania 2023 5%; Uganda 2020 7·1%; Lesotho 2020 28%; Eswatini 2021 32%; Malawi 2020 10%; Namibia 2017 15%; Zambia 2021 13%; Cameroon 2018 3%; Côte d’Ivoire 2017–18 4%; Rwanda 2019 3·3%; Kenya 2018† 6·6%; South Africa 2022 16%
HIV prevalence in men aged 15–49 years	7·7% (2·5–24·4)	Zimbabwe 2020 9%; Tanzania 2023 2%; Uganda 2020 3·8%; Lesotho 2020 16%; Eswatini 2021 16%; Malawi 2020 6%; Namibia 2017 8%; Zambia 2021 6%; Cameroon 2018 2%; Côte d’Ivoire 2017–18 1%; Rwanda 2019 1·8%; Kenya 2018† 3·1%; South Africa 2022 9%
HIV incidence per 100 person-years in women aged 15–49 years	0·70 (0·17–4·05)	Malawi 2016 0·44, 2020 0·31; Zambia 2021 0·63; Zimbabwe 2020 0·67; Lesotho 2020 0·81; Eswatini 2021 1·45; Tanzania 2023 0·29; Cameroon 2017 0·40; Rwanda 2019 0·07; Uganda 2020 0·42; Kenya 2018† 0·17; South Africa 2022 0·87
HIV incidence per 100 person-years in men aged 15–49 years	0·42 (0·11–1·80)	Malawi 2016 0·22, 2020 0·15; Zambia 2021 0·05; Zimbabwe 2020 0·23; Lesotho 2020 0·33; Eswatini 2021 0·20; Tanzania 2023 0·12; Cameroon 2017 0·08; Rwanda 2019 0·09; Uganda 2020 0·21; Kenya 2018† 0·13; South Africa 2022 0·64
Percentage of women with HIV diagnosed	92% (83–97)	Malawi 2020 90%; Zambia 2021 90%; Zimbabwe 2020 88%; Namibia 2017† 90%; Tanzania 2017 55%, 2023 85%; Ethiopia† 2018 83%; Côte d’Ivoire 2017–18† 43%; Cameroon 2017† 58%; Mozambique 2021 73%; Uganda 2021 84%; Rwanda 2019 86%; Eswatini 2021 95%; Lesotho 2020 91%; Kenya 2018† 83%; South Africa 2022 92%
Percentage of men with HIV diagnosed	83% (72–93)	Malawi 2020 85%; Zambia 2021 87%; Zimbabwe 2020 84%; Namibia 2017† 80%; Tanzania 2017 45%, 2023 78%; Ethiopia† 2018 70%; Côte d’Ivoire 2017–18† 24%; Cameroon 2017† 51%; Mozambique 2021 69%; Uganda 2021 76%; Rwanda 2019 80%; Eswatini 2021 92%; Lesotho 2020 88%; Kenya 2018† 73%; South Africa 2022 85%
Percentage of women diagnosed with HIV on ART‡	96% (90–98)	Lesotho 2020 98%; South Africa 2022 91%; Eswatini 2021 98%; Namibia 2017† 97%; Zambia 2021 98%, Tanzania 2023 98%; Ethiopia 2018† 96%; Malawi 2020 98%; Uganda 2021 97%; Cameroon 2017† 93%; Zimbabwe 2020 98%; Côte d’Ivoire 2017–18† 93%; Mozambique 98%; Rwanda 98%; Kenya 2018 97%
Percentage of men diagnosed with HIV on ART‡	95% (87–98)	Lesotho 2020 96%; South Africa 2022 90%; Eswatini 2021 96%; Namibia 2017† 95%; Zambia 2021 98%; Tanzania 2023 97%; Ethiopia 2018† 99%; Malawi 2020 97%; Uganda 2021 95%; Cameroon 2017† 94%; Zimbabwe 2020 96%; Cote d’Ivoire 2017–18† 71%; Mozambique 94%; Rwanda 97%; Kenya 2018 95%
Percentage of women on ART with viral load less than 1000 copies per mL‡	95% (84–98)	Zambia 2021 96%; Malawi 2020 97%; Zimbabwe 2020 91%; Namibia 2017 92%; Tanzania 2023 95%; Ethiopia 2018† 86%; Côte d’Ivoire 2017–18† 78%; Cameroon 2017 80%; Mozambique 2021 90%; Uganda 2021 93%; Rwanda 2018 92%; Eswatini 2021 96%; Lesotho 2020 92%; Kenya 2018 90%; South Africa 2022 94%
Percentage of men on ART with viral load less than 1000 copies per mL†	93% (81–97)	Zambia 2021 97%; Malawi 2020 97%; Zimbabwe 2020 89%; Namibia 2017 90%; Tanzania 2023 93%; Ethiopia 2018† 91%; Côte d’Ivoire 2017–18† 65%; Cameroon 2017 81%; Mozambique 2021 88%; Uganda 2021 91%; Rwanda 2018 85%; Eswatini 2021 98%; Lesotho 2020 90%; Kenya 2018 91%; South Africa 2022 94%
Percentage of all people positive for HIV with viral load less than 1000 copies per mL	78% (64–88)	Zambia 2021 86%; Malawi 2020 87%; Zimbabwe 2020 76%; Eswatini 2021 87%; Lesotho 2020 81%; Tanzania 2023 78%; Uganda 2020 75%; Namibia 2017† 77%; Ethiopia 2018† 70%; Côte d’Ivoire 2017–18† 40%, Cameroon 2017† 47%; Rwanda 2019 76%; Kenya 2018 72%
Prevalence of unsuppressed HIV (percentage of all adults aged 15 years or older with HIV and viral load more than 1000 copies per mL)	2·7% (0·9–9·8)	Zambia 2021† 1·4%; Namibia 2017† 2·8%; Malawi 2020 1·2%; Zimbabwe 2020 (aged 15 years or older) 3·1%; Côte d’Ivoire† 2018 1·7%; Eswatini 2021 3·2%; Lesotho 2020 4·3%; Uganda 2020 1·4%; Kenya 2018 1·4%
Proportion of people aged 15–64 years with an indication for PrEP§	5·2% (1·7–16·6)	Equivalent data not available; Rwanda 2018–19 4% of women and 16% of men, Zimbabwe 2018–19 4% of women and 19% of men, Lesotho 2020 7% of women and 25% of men, Malawi 2020 3% of women and 20% of men, Uganda 2020–21 4% of women and 24% of men reported more than 2 sexual partners (not necessarily condomless) in past 12 months

Model outputs are median (90% range). Restricted to setting-scenarios with HIV incidence in 2024 of more than 1 per 100 person-years. We show national data from countries, but setting-scenarios also reflect subsettings within countries as well as countries as a whole. ART=antiretroviral therapy. PrEP=pre-exposure prophylaxis.

* All observed data are from Population Health Impact Assessments surveys apart from data for South Africa, which are from The Sixth South Africa National HIV, Incidence and Behaviour Survey.

† Aged 15–64 years.

‡ Adjusted for having a detectable antiretroviral in blood.

§ Indication for PrEP is defined by condomless sex in past 3 or 6 months with a new partner.

**Table 2:** Predicted outcomes over 10 years of the three policies across setting-scenarios*

	No DCP	DCP without cabotegravir	DCP including cabotegravir
Proportion of women aged 15–49 years who tested for HIV in the past year	24% (11–58)	25% (12–59)	28% (13–60)
Proportion of men aged 15–49 years who tested for HIV in the past year	8% (4–36)	10% (5–37)	12% (5–38)
Proportion of people aged 15–64 years with an indication for PrEP or DCP	5% (2–18)	5% (2–18)	5% (2–17)
Proportion of people with an indication for PrEP who were under DCP	NA	20% (10–31)	39% (16–52)
Proportion of people with a PrEP indication who were on PrEP	4% (1–11)	15% (6–25)	33% (10–46)
Proportion of people aged 15–64 years who were on PrEP	0·2% (0–1·0)	0·8% (0·1–3·4)	1·7% (0·2–6·6)
Proportion of people with a PrEP indication and a HIV test in the past 3 months who were on PrEP	14% (4–43)	54% (23–74)	71% (35–83)
Proportion of people with an indication for PrEP or DCP and were on PrEP in the past 3 months who remain on PrEP	67% (30–86)	91% (87–95)	93% (90–96)
Proportion of people with an indication for PrEP or DCP and were on PrEP in the past 3 years† who are still on PrEP	55% (19–83)	82% (67–93)	85% (70–94)
Proportion of people who were under DCP and continued to have an indication for PrEP or DCP who dropped off from DCP in past 3 months	NA	2·6% (0·8–7·6)	1·6% (0·5–4·4)
Proportion of people who were under DCP 3 months ago‡ who dropped off from DCP	NA	30% (16–44)	27% (15–41)
Proportion of people on DCP who had an indication for PrEP	NA	68% (53–83)	69% (55–84)
Proportion of people on PrEP on long-acting cabotegravir	0	0	77% (68–81)
Proportion of people on oral PrEP with adherence >80%§	62% (0–89)	62% (0–88)	63% (0–89)
Proportion of people who were HIV positive with integrase inhibitor resistance	2·7% (0·7–10·3)	2·7% (0·7–10·3)	3·2% (1·1–11·5)

Data are median (90% range). DCP=dynamic choice HIV prevention. PrEP=pre-exposure prophylaxis. NA=not applicable.

* For each setting-scenario we calculate the mean over the all 3-month periods in the 10 years.

† Not including the current period.

‡ Might not necessarily still have an indication for PrEP or DCP.

§ Hair tenofovir concentrations in the DCP trial showed the drug was present in 70% of people reporting use of PrEP.

**Table 3:** Predicted effects on HIV incidence, prevalence, and care cascade measures over 10 years and 1000 setting-scenarios

	No DCP	DCP without cabotegravir	DCP including cabotegravir
HIV incidence per 100 person years (aged 15–49 years)	0·51 (0·12–2·61)	0·45 (0·11–2·38)	0·32 (0·09–1·71)
HIV incidence rate ratio *vs* continue with current policies	NA	0·89 (0·67–1·17)	0·64 (0·44–0·97)
Total number of new infections over 10 years in adults aged 15–64 years	466 000 (122 000–1 880 000)	416 000 (110 000–1 710 000)	306 000 (89 000–1 232 000)
Percentage of people positive for HIV who are diagnosed	90% (82–96)	91% (84–96)	92% (86–97)
Percentage of people positive for HIV with viral load less than 1000 copies per mL	80% (67–89)	81% (68–89)	82% (70–90)
Prevalence of adults with HIV viral load more than 1000 copies per mL	2·4% (0·7–9·0)	2·2% (0·7–8·5)	1·9% (0·6–7·3)
HIV prevalence (aged 15–49 years)	9·6% (2·8–32·5)	9·3% (2·8–32·5)	8·9% (2·7–30·1)
Percentage of children born to mothers with HIV who have HIV by the end of the breastfeeding period	6·3% (2·5–14·5)	6·0% (2·4–14·0)	5·8% (2·3–13·6)

Data are median (90% range). DCP=dynamic choice HIV prevention. PrEP=pre-exposure prophylaxis.

**Table 4:** Breakdown of discounted annual costs over 50 years to 2075 and undiscounted annual costs over 5 years to 2030

	No DCP		DCP without cabotegravir		DCP including cabotegravir	
	Discounted annual costs over 50 years ($ millions)	Undiscounted annual costs over 5 years ($ millions)	Discounted annual costs over 50 years ($ millions)	Undiscounted annual costs over 5 years ($ millions)	Discounted annual costs over 50 years ($ millions)	Undiscounted annual costs over 5 years ($ millions)
DCP visits*	0	0	7·6	7·3	13·9	13·0
HIV testing	9·0	15·4	9·9	16·1	12·1	18·1
Oral PrEP or PEP drug	1·2	1·5	5·1	4·9	1·7	2·2
Cabotegravir PrEP drug	0	0	0	0	8·3	7·5
PrEP clinic visits	0·8	1·0	3·4	3·3	9·4	9·0
ART drug	51·4	78·9	49·0	79·0	42·8	79·0
Cotrimoxazole	4·4	7·1	4·2	7·1	3·6	7·1
ART clinic visits	24·0	38·4	22·8	38·5	19·7	38·5
Viral load tests	13·1	20·7	12·4	20·7	10·5	20·7
CD4 cell count tests	0·9	1·5	0·9	1·5	0·7	1·4
HIV-related clinical care costs	14·7	21·6	14·0	21·5	12·6	21·2
VMMC	1·7	5·1	1·8	5·2	1·9	5·3
Care for children with HIV	8·8	14·1	7·9	13·5	6·2	12·3
Total	130·2	205·3	138·8	218·5	143·5	235·3

Data are millions (US$). DCP=dynamic choice HIV prevention. PrEP=pre-exposure prophylaxis. PEP=postexposure prophylaxis. VMMC=voluntary medical male circumcision.

* Beyond costs of PrEP or HIV testing.

**Table 5:** HIV-related deaths, DALYs, and costs over 50 years and cost-effectiveness analysis compared with no DCP

	No DCP	DCP without cabotegravir	DCP including cabotegravir
Difference in number of HIV-related deaths per year	··	−1300 (−1400 to −1200); −4500 to 800	−3700 (−4000 to −3400); −12 300 to 200
Difference in DALYs per year over 50 years	··	−18 400 (−20 100 to −16 700); −68 600 to 19 000	−56 400 (−60 500 to −52 300); −178 800 to 8200
Difference in annual cost over 50 years	··	$8·6 million (7·7 to 9·4); −6·1 to 32·3	$13·2 million (11·6 to 14·8); −16·9 to 61·0
Difference in net DALYs* per year over 50 years	··	−1200 (−3600 to 1200); −65 900 to 55 800	−29 900 (−35 400 to −24 400); −179 700 to 81 700
Incremental cost-effectiveness ratio (cost per DALY averted)	··	Dominated	$234
Incremental cost-effectiveness ratio (cost per DALY averted) for DCP compared with no DCP (if long-acting cabotegravir PrEP not available)	··	$467	NA
Percent of setting-scenarios for which policy incurs the lowest DALYs	6%	11%	83%
Percent of setting-scenarios for which policy has the lowest cost	69%	8%	23%
Percent of setting-scenarios for which policy has the lowest net DALYs (ie, it is the cost-effective policy)	30%	14%	56%
Incremental cost-effectiveness ratio (cost per DALY averted) according to cost of DCP visits			
$5	··	Dominated	$90
$10	··	Dominated	$194
$12†	··	Dominated	$234
$15	··	Dominated	$297
$20	··	Dominated	$400
$25	··	Dominated	$503
Incremental cost-effectiveness ratio (cost per DALY averted) according to cost of long-acting cabotegravir PrEP per year			
$20	··	Dominated	$137
$40	··	Dominated	$186
$60†	··	Dominated	$234
$80	··	Dominated	$284
$200	··	$467	$632
Total annual cost of DCP including cabotegravir‡			
$130	··	Dominated	$88
$190†	··	Dominated	$234
$330	··	$467	$631

Data are mean (95% CI); 90% range, unless otherwise stated. Monetary values are US$. DCP=dynamic choice HIV prevention. PrEP=pre-exposure prophylaxis. DALY=disability-adjusted life-year.

* Given a cost-effectiveness threshold of $500 per DALY averted.

† Value used in main analysis.

‡ In the primary analysis, the value used was $190, which includes $15 per 3 months for long-acting cabotegravir ($60 per year), $15 per 3 months for long-acting cabotegravir injection administration (ie, visit cost; $60 per year), $12 for a DCP visit ($48 per year), and $22 for six HIV tests per year. The value of $130 was based on halving the cost of long-acting cabotegravir and injection administration. The value of $330 was based on annual cost of long-acting cabotegravir of $200.

**Table 6:** Sensitivity analyses by annual cost of DCP including cabotegravir

	<4% of people aged 15–64 years with an indication for PrEP				4–7% of people aged 15–64 years with an indication for PrEP				≥7% of people aged 15–64 years with an indication for PrEP			
	ICER	Percentage of setting-scenarios ICER is below cost-effectiveness threshold			ICER	Percentage of setting-scenarios ICER is below cost-effectiveness threshold			ICER	Percentage of setting-scenarios ICER is below cost-effectiveness threshold		
		$300	$500	$1000		$300	$500	$1000		$300	$500	$1000
**$130**												
Prevalence of unsuppressed HIV												
<2·0%	$144	59%	64%	71%	$483	37%	49%	66%	$2286	8%	11%	30%
2–3·99%	$83	86%	89%	93%	$111	68%	77%	88%	$597	23%	40%	62%
≥4·0%	Cost-saving	100%	100%	100%	Cost-saving	96%	96%	99%	$40	82%	90%	96%
**$190 (used in primary analysis)**												
Prevalence of unsuppressed HIV												
<2·0%	$325	48%	57%	66%	$757	22%	37%	57%	$3112	3%	9%	16%
2–3·99%	$19	76%	83%	89%	$269	50%	67%	83%	$925	12%	24%	46%
≥4·0%	Cost-saving	100%	100%	100%	$38	91%	96%	97%	$164	64%	81%	92%
**$330**												
Prevalence of unsuppressed HIV												
<2·0%	$745	31%	42%	59%	$1397	11%	17%	39%	$5060	2%	3%	9%
2–3·99%	$257	58%	68%	82%	$639	20%	36%	68%	$1688	2%	4%	23%
≥4·0%	Cost-saving	91%	96%	100%	$146	66%	82%	96%	$454	34%	52%	81%

Monetary values are US$. ICER for DCP including cabotegravir (compared with no DCP) according to the proportion of people with an indication for PrEP, the prevalence of unsuppressed HIV in 2024, and the annual cost of DCP including cabotegravir. In the primary analysis the value used was $190—this included $15 per 3 months for long-acting cabotegravir drug ($60 per year), $15 per 3 months for long-acting cabotegravir injection administration (ie, visit cost; $60 per year), $12 for a DCP visit ($48 per year), and $22 for six HIV tests per year. The value of $130 was based on halving the cost of long-acting cabotegravir drug and injection administration. The value of $330 was based on the annual cost of long-acting cabotegravir drug of $200. DALY=disability-adjusted life-year. DCP=dynamic choice HIV prevention. ICER=incremental cost-effectiveness ratio. PrEP=pre-exposure prophylaxis.

## Data Availability

The model code used for this paper is available at https://figshare.com/articles/software/Code_for_project_Dynamic_Choice_HIV_Prevention_in_the_context_of_Injectable_Cabotegravir_CAB-LA_PrEP_a_model-based_cost-effectiveness_analysis/29204546.

## Research in context

### Evidence before this study

Models have shown that provision of pre-exposure prophylaxis (PrEP), including long-acting PrEP and oral PrEP, in settings in east, central, south, and west Africa has the potential to lead to reductions in HIV incidence and be a cost-effective intervention in specific circumstances. A dynamic choice HIV prevention (DCP) intervention has been shown to lead to substantially increased coverage of PrEP and post-exposure prophylaxis (PEP) use and to reduce incident HIV infections. We searched Web of Science from database inception to May 31, 2025, using the search terms “hiv AND choice AND prevention AND cost AND effect* AND model*”, restricted to the English language, and we found no papers looking at the cost-effectiveness of this specific DCP intervention.

### Added value of this study

We found that the DCP intervention was likely to be cost-effective in multiple settings should it be possible to source long-acting drugs at a sufficiently low cost. In addition to the cost of DCP plus long-acting cabotegravir PrEP and the choice of cost-effectiveness threshold, the prevalence of unsuppressed HIV and the proportion of people with an indication for PrEP are both key factors when assessing whether the intervention is cost-effective in a given setting.

### Implications of all the available evidence

Offering structured PrEP and PEP choices including long-acting cabotegravir, enabling risk-informed use, could reduce HIV incidence by a third over 10 years. If projected generic production costs of long-acting cabotegravir can be realised it is likely to be cost-effective across multiple settings in east, central, south, and west Africa.
